# A One-*Versus*-All Class Binarization Strategy for Bearing Diagnostics of Concurrent Defects

**DOI:** 10.3390/s140101295

**Published:** 2014-01-13

**Authors:** Selina S. Y. Ng, Peter W. Tse, Kwok L. Tsui

**Affiliations:** Department of Systems Engineering and Engineering Management (SEEM), City University of Hong Kong, Tat Chee Avenue, Kowloon, Hong Kong, China; E-Mails: meptse@cityu.edu.hk (P.W.T.); kltsui@cityu.edu.hk (K.L.T.)

**Keywords:** bearing, multiple defects, fault diagnostics, class binarization, support vector machine (SVM), decision tree

## Abstract

In bearing diagnostics using a data-driven modeling approach, a concern is the need for data from all possible scenarios to build a practical model for all operating conditions. This paper is a study on bearing diagnostics with the concurrent occurrence of multiple defect types. The authors are not aware of any work in the literature that studies this practical problem. A strategy based on one-*versus*-all (OVA) class binarization is proposed to improve fault diagnostics accuracy while reducing the number of scenarios for data collection, by predicting concurrent defects from training data of normal and single defects. The proposed OVA diagnostic approach is evaluated with empirical analysis using support vector machine (SVM) and C4.5 decision tree, two popular classification algorithms frequently applied to system health diagnostics and prognostics. Statistical features are extracted from the time domain and the frequency domain. Prediction performance of the proposed strategy is compared with that of a simple multi-class classification, as well as that of random guess and worst-case classification. We have verified the potential of the proposed OVA diagnostic strategy in performance improvements for single-defect diagnosis and predictions of BPFO plus BPFI concurrent defects using two laboratory-collected vibration data sets.

## 1. Introduction

In bearing diagnostics using a data-driven modeling approach, a concern is the need for data from all possible scenarios to build a practical model for all operating conditions. This paper is a study on bearing diagnostics with the concurrent occurrence of multiple defect types. The authors are not aware of any work in the literature that studies this practical problem. In this paper, a strategy based on one-*versus*-all (OVA) class binarization is proposed to improve fault diagnostics accuracy while reducing the number of scenarios for data collection, by predicting concurrent defects from training data of normal and single defects. The purpose of this procedure is to develop an effective bearing diagnostic model considering the possibility of concurrent occurrence of multiple defects. It will help build more practical classifiers with less data, for improved performance under more operating conditions. These help provide advanced failure warning and reduce unexpected failures in real applications.

This paper is a study on vibration-based bearing diagnostics with the data-driven approach. Data mining and knowledge discovery methods are used, with consideration of non-exclusive bearing defect types. The problem of bearing fault diagnostics is formulated to diagnose multiple defects from normal and single defect training data. The accuracy of the proposed OVA strategy on bearing diagnosis of unseen observations is evaluated with vibration data collected from laboratory.

The remaining of this section is a brief review on bearing diagnostic analysis of acceleration signals obtained from piezoelectric sensors, using data mining methods. Section 2 is a brief summary on the background of techniques used in this paper, namely support vector machine (SVM); C4.5 decision tree; and class binarization. Section 3 introduces the hypothesis and logic behind the problem formulation used in modeling. Empirical analyses of two bearing data sets, collected from a bearing fault motor and a multiple bearing mechanical system, are reported in Sections 4 and 5 respectively, each followed by a discussion of the results in the same section. Finally, this paper closes with the conclusions in Section 6.

### 1.1. Bearing Fault Diagnostics

Bearings are one of the most widely used components in machines and bearing failure is one of the most frequent reasons for machine breakdown. According to [1], almost 40%–50% of motor failures are bearing-related. Bearing fault diagnostics has a long history in research [2–4] and vibration analysis has been established as the most common and reliable method in this context [5–7]. The literature concerning the use of acceleration signals obtained from piezoelectric sensors in bearing diagnostics is so huge that a review is not the most useful format in summarizing recent research developments. Below is an overview of the logic behind vibration-based bearing fault diagnostics and some literature highlights of two approaches to the analysis, with focus on the statistical and data mining approach.

Bearing faults can refer to localized defects or extended spalls. In rotating machinery, whenever a defect makes contact with another surface, a high-level short-duration vibration impulse is excited. The effect is a damped signal which comprises of a sharp rise corresponding to the impact and approximately exponential decay [8,9]. While the decay limits the time window that a signal can be detected, the sharp rise at the leading edge allows the identification of the impact time and hence time between impacts. The occurrence of impulses is pseudo-cyclostationary [10], *i.e.*, they can be modeled as cyclostationary, although in reality the impulse due to a particular fault may not be excited in every cycle of rotation.

Since the rotation of the machinery is periodic, impulses and the damped signals due to a particular defect also tend to be generated periodically. The frequency of impulse occurrence is the characteristic defect frequency of a particular defect and the theoretical value can be calculated from the physics of a component. The existence of a fault is indicated by the occurrence of the impulses at the characteristic defect frequency, and the impulse amplitude provides some information about how serious the fault is. The same information also occurs as sidebands in the high resonant frequency bands.

The goal in fault detection and diagnosis is to extract from vibration signals the presence of impulses and identify the matching characteristic defect frequencies, which act as fault signatures for the defects concerned. In practice, vibration signals due to defect impacts mix together with those generated by normal rotation of the same component, other components of the same rotating machinery and other random vibrations. Another complication is that the actual frequency is affected by the tightness-of-fit of the components and there are generally some differences between the measured frequencies and the theoretical characteristic defect frequency values due to random slip [8].

#### 1.1.1. Approaches in Bearing Fault Diagnostics

The signal processing approach constitutes a major part of the literature in vibration-based bearing fault diagnostics. This approach has been developed for decades with a huge literature and is not the focus of this paper. A useful review in this classic approach is given in [11], which summarizes various topics in bearing condition monitoring, from the underlying science to signal processing techniques that can be used with signals from accelerometers. More recently, the research developments since 1969 under the classic signal processing approach have been summarized in a tutorial overview [10], where a procedure that is useful in the majority of cases is also described. Some popular techniques in the signal processing approach for bearing fault diagnostics include fast Fourier transform (FFT), wavelet methods, spectral kurtosis (SK), amplitude demodulation by envelope analysis or Hilbert transform, described in papers such as [12–14].

Another approach to vibration-based bearing diagnostics is the use of data mining techniques. Data mining, first appeared as knowledge discovery in databases [15], and emerged from a diverse background of databases, statistics and machine learning [16,17]. Another related research field is pattern recognition, emerged from engineering disciplines, which can be viewed as another facet of the same field as machine learning, emerged from computer science [18]. According to Bishop, the field of pattern recognition is concerned with the automatic discovery of regularities in data through the use of computer algorithms. In contrast, the multi-disciplinary research field of data mining focuses on the non-trivial extraction of implicit, previously unknown, potentially useful knowledge about data [19,20]. Their advantages over signal processing methods include better adaptation to complex systems; better accommodation to uncertainty; and the fact they do not require calculation of characteristic defect frequencies from the physics of the components. On the other hand, the use of these methods requires the collection of sufficient historical data for the training of models, enabled by the fast development of small size, low cost sensors.

Data mining techniques are grouped under different names in the machine diagnostics literature. For example, in the review on machinery diagnostics and prognostics [21], Jardine *et al.* summarized the diagnostics techniques into statistical approaches which included hypothesis test; cluster analysis; support vector machine (SVM), hidden Markov model (HMM), and artificial intelligence approaches which included artificial neural network (ANN), expert systems (ES), fuzzy systems, and evolutionary algorithms such as genetic algorithms (GA). In a more recent overview [22], data mining techniques used in mechanical systems research were grouped under the term natural computing if they were motivated or suggested by biological systems or processes. In this overview, ‘traditional’ probability and statistics such as hypothesis tests were omitted. They discussed in detail some data mining methods such as ANN, fuzzy sets and fuzzy logic, statistical learning theory and kernel methods such as SVM and relevance vector machine (RVM), simulated annealing, GA, Gaussian process, graphical methods and deep belief networks. Other data mining techniques mentioned in this paper include distance-based classifiers, Bayesian methods, clustering, decision trees, and HMM. These methods are also referred to as ‘intelligent’ bearing fault diagnostics in some papers [23,24]. Data mining techniques applied to vibration-based bearing diagnostics include Bayesian inference [25], HMM [26], hidden semi-Markov model [27], SVM [28], ANN and GA [29], PCA and decision tree [30]. Important aspects in successful modeling with statistical and data mining techniques are also discussed under the context of vibration-based bearing diagnostics. For example, fault feature extraction from bearing accelerometer sensor signals is discussed in [31–33]. The effect of number of features used in bearing fault diagnostics with SVM and proximal support vector machine (PSVM) is discussed in [34]. Like signal processing methods, statistical and data mining methods have been used in fault detection, diagnostics and prognostics of various machinery components. More extensive survey of these methods can be found in the machine prognostics literature such as [35,36].

#### 1.1.2. Fault Diagnostic Systems for Industrial Applications

No matter which approach is used in the fault diagnosis, some common requirements exist for an ideal fault diagnostic system. While the discussion in [37] focused on fault diagnostics of chemical processes, the desirable attributes they listed for a fault diagnostic system could also be applied in machinery diagnostics:

1. Early detection and diagnosis ability: Achieve a balance in the trade-off between quick response and high false alarm rate.
2. Fault isolation ability: Be able to discriminate between different failures at different locations and different levels.
3. Robustness: To maintain performance at an acceptable level under noise and uncertainty.
4. Novelty identification ability: The ability to decide the state of a system as normal or abnormal, and if abnormal, a known fault or a novel fault state.
5. Multiple fault identification ability: The ability to identify multiple faults, which is difficult due to the interacting nature of most faults.
6. Adaptability: The ability to adapt to changes in external inputs or structural changes.
7. Reasonable storage and computational requirement: Achieve a balance in the trade-off between the computational complexity and system performance.
8. Explanation facility: To reason about cause and effect relationship in a process and provide possible explanations on how the fault originated and propagated to the current situation. To justify why certain hypotheses are proposed and why certain others are not, thus help the operator evaluate and act upon his/her experience.

The focus of this paper lies in the multiple fault identification ability with statistical and data mining techniques. One or more of different defect types can occur on a bearing concurrently in practice. A bearing diagnostic method that is able to identify both single and multiple defects is more useful in real-world applications. In classification, training data for all system states to be recognized by a model are usually necessary [38]. Researchers in the classic signal processing approach [10] are also concerned on the need of a large amount of data of all permutations and combinations of different scenarios in the data-driven approach. This paper is a study to propose ways of reducing the number of scenarios for data collection, on different combination of defect types, while maintaining or even improving the diagnostic performances. Two popular statistical and data mining methods, namely SVM and C4.5, are used in this study.

The proposed OVA approach is designed for fault diagnosis in the second phase of the systems health management process. In a real application such as machinery fault diagnostics and prognostics in a factory, the first phase *i.e.*, the initial establishment of the process involves identifying how many and which critical components to monitor, based on expert knowledge; maintenance history; costs and budget. These are out of the scope of this paper. The second phase of the process, which concerns the regular production use of the process, involves the fault detection; isolation; diagnosis; prognosis and remaining useful life prediction; and maintenance decisions. During the transition period before samples of all fault types are collected, the fault detection and isolation can still be performed. The faulty component/structure can then be removed and examined by domain expert for diagnosis, and the data sample can be added to the training data with the assigned fault type if considered appropriate by the domain expert. The proposed OVA approach helps speed up this process by eliminating the need of data collection of all different types of concurrent faults of a component/structure.

## 2. Technique Overview

Algorithms in data mining are often grouped by the type of model generated or the most usual way of formulating a problem. Table 1 lists the most popular data mining algorithms, along with their model types and their original problem formulations.

Classification and regression are the most common data mining formulations, where the classes (categorical in classification and continuous in regression) are utilized in the modeling (supervised learning). Five of the ten popular algorithms are designed for classification and regression.

The most intuitive formulation for fault diagnostics is classification and the simplest formulation for fault prognostics is regression. As in any modeling problems, the actual formulation used in the diagnosis/prognosis depends on the data available and how the analyst formulates the problem. For example, bearing diagnostics can also be modeled as a clustering problem [40] for unsupervised learning. Bearing remaining useful life can be predicted by classification [41], which can be considered as an approximation to simplify the modeling, when difference of a few minutes does not matter in the prognosis interpretation. This allows a wide range of classification techniques to be applied to the prognostics problem. Diagnosis with a regression method [42] is also computationally possible, though there is no physical meaning to a decimal regression estimate in between different defect types.

In this paper, we stick to the classic formulation of bearing diagnosis with classification. Classification, the most familiar and most popular data mining task, is a mapping from the database to the set of predefined, non-overlapping classes that partition the entire database. SVM is a kernel method which builds a model by constructing a hyperplane that best separates two classes. Decision tree methods recursively generate a tree by constructing hierarchical and non-overlapping classification rules using a greedy algorithm. By taking the best immediate, or local, solution in finding an answer, the algorithm selects an attribute to split on at any given node with any given data set, decides whether to stop branching and what branches to form. SVM and C4.5 are used in our proposed method due to its popularity in prognostics and systems health management (PHM) related research, including but not limited to bearing diagnostics with vibration data.

Besides the algorithm(s) to use, the way how data are utilized in modeling is another critical factor for the success of data-driven methods in modeling a problem. For example, when a multi-class real-world problem is modeled with a binary class classification algorithm, as in SVM, different class binarization strategies can be used. Just as classification algorithms can be useful in regression problems, class binarization strategies may also be utilized in improving diagnostics performances and possibly enable diagnosis of multiple defects from single defect training data.

The following notations will be used in both Sections 2.1 and 2.2. Let **X** be the *n*-by-*d* matrix of predictors used in training and *Y* be the *n* vector of corresponding classes (responses) assigned to each example (instance) in the training set *D*, where *n* is the number of training examples and *d* the number of predictors (features).The rest of this section is a brief overview of the SVM algorithm; the C4.5 decision tree and the class binarization strategy to be used in the proposed method.

### 2.1. SVM

SVM is a kernel method first introduced in [43], which builds a model by constructing a hyperplane that best separates two classes. A detail review of the algorithms, including the SVM for classification and the support vector regression (SVR) for regression can be found in [44]. A more concise introduction in the context of structural health management can be found in [45].

Consider the case of two classes, *i.e.*, *Y* ∈ {1, −1}. When the two classes are linearly separable, it is possible to define a separating hyperplane *f*(**X**) = 0, characterized by the *n*-vector ***w*** and the scalar *b*:

(1) $$f(X)=w^{T}x+b=\sum_{i=1}^{n}w_{i}x_{i}+b=0$$

If the two classes are completely separable, the data set should satisfy the constraints below:

(2) $$\{\begin{array}{c} f\left(x_{i}\right)=1\;\text{if}\;y_{i}=1\\ f\left(x_{i}\right)=-1\;\text{if}\;y_{i}=-1 \end{array}$$

These can alternatively be expressed in complete equation,

(3) $$y_{i}f\left(x_{i}\right)=y_{i}(w^{T}x_{i}+b)\geq 1\;\text{for}\;i=1,2,\ldots ,n$$

where the geometrical margin between the two classes is 2/‖**w**‖.

The optimal separating hyperplane is the separating hyperplane with greatest distance between the plane and the nearest data points on both sides, *i.e.*, the boundary in the middle of the maximum geometrical margin between the two classes. The nearest data points that used to define the margin are called support vectors. After the support vectors are selected, the rest of features are not required.

With consideration of the not completely separable case, the SVM modeling can be expressed as an optimization problem with slack variables *ξ_i_* and error penalty C:

(4) $$\begin{array}{c} \text{Minimize}\;\frac{1}{2}{∥\text{w}∥}^{2}+C\sum_{i=1}^{n}\xi_{i}\\ \text{Subject to}\;\{\begin{array}{c} y_{i}({\text{w}}^{T}x_{i}+b)\geq 1-\xi_{i}\\ \xi_{i}\geq 0 \end{array}\;\text{for}\;i=1,2,\ldots ,n \end{array}$$

where *ξ_i_* measures the distance between a data point *x_i_* and the boundary, for any *x_i_* that lies on the wrong side of the margin. To simplify the calculation, the above optimization problem can be converted to the equivalent Lagrangian dual problem with Kuhn-Tucker condition, followed by the substitution of the discrete form of saddle-point equations to obtain the dual quadratic optimization problem.

For non-linear classification tasks, the application of kernel functions is required to transform the problem into a higher dimensional feature space to make linear separation possible. The choice of the appropriate kernel function is very important. A common choice is a polynomial kernel with the kernel parameters and the penalty margin *C* selected by cross-validation.

For problems with more than two classes, a multi-class classification strategy, such as one-*versus*-all (OVA), one-*versus*-one (OVO), or directed acyclic graph (DAG), is required.

Recent examples of research papers applying SVM to bearing fault diagnostics include [46–48]. SVM methods have also been applied in other bearing PHM formulations such as one-class anomaly detection [49], two-class fault detection [50], prognostics and RUL prediction [51]. In particular, Widodo and Yang [46] summarized the use of SVM in machine condition monitoring and fault diagnosis up to 2007 into a table, categorized by machine component, with the multi-class handling strategy used if specified in the corresponding literature. Abbasion *et al.* [47] discussed multi-fault diagnosis of normal and three fault types in two different bearings at different locations of a system. Sugumaran *et al.* [48] discussed the use of a multi-class SVM built from one-class SVMs to reduce the computational efforts required by the traditional binary SVM. The authors are not aware of any publications in the literature that discuss the prediction of combined-fault conditions from normal and single-fault training data. In this paper, we propose a novel strategy to accomplish this by OVA class binarization.

The SVM used in this paper is built using sequential minimal optimization (SMO) [52]. Although a SVM model depends on only a subset of the training data, it is well-known for its excellent performance in generalization and the classification of high-dimensional data [46]. It also has a reputation for handling dependent classes. However, just like any methods, SVM methods may not be the most accurate algorithm for all situations. In particular, it does not naturally handle multiple classes or generate proper probability estimates. In the formal statistical sense, the SVM do not belong to techniques of the ‘Bayesian’ approach. While historical data are used to train the models, no notion of subjective probability is introduced thus these two methods do not require updating of priors. An example of a ‘Bayesian’ technique is the relevance vector machine (RVM) [53].

### 2.2. C4.5 Decision Tree

C4.5 decision tree is the most popular method in the ID3 decision tree family. The ID3 decision tree [54] family chooses attributes on the basis of information gain (reduction in entropy). C4.5 is an enhancement of ID3 that constructs a branch using the attribute with maximum information gain ratio.

In Information theory, a probabilistic method for quantifying information is developed by Shannon [55]. The entropy *H(Y)* of the distribution *p(y)*, a measure of the uncertainty about *Y*, is defined as:

(5) $$H\left(Y\right)=-\sum_{i=1}^{n}p(y_{i})\log(y_{i})$$

The entropy ranges from zero to infinity. It is zero if and only if there is only one probable value for *Y* (with probability 1) and all other values have zero probability. Its value is at its maximum if and only if all possible values for *Y* are equally probable.

Similarly, the uncertainty of *Y* with knowledge on *X_j_*, or the conditional entropy *H*(*Y*∣*X_j_*) on the distribution of the conditional probability *p*(*y*∣*x_j_*), is given by:

(6) $$\begin{array}{c} H\left(Y|X_{j}\right)=-\sum_{j=1}^{d}\sum_{i=1}^{n}p\left(y_{i}|x_{j}\right)\log\left(y_{i}|x_{j}\right)\\ =-\sum_{v\in \chi }\frac{\left|D_{x_{j}=v}\right|}{\left|D\right|}H(Y|x_{j}=v) \end{array}$$

where χ is the set of possible values of *X_j_*, *D_xj_*_=_ *_v_* is the subset of the dataset for which feature *x_j_* has value *v*, and the notation | • | denotes the number of instances in a dataset.

Information gain Gain (*Y*, *X_j_*), or average mutual information *I*(*X_j_*; *Y*), is a measure of reduction in uncertainty about *Y* due to the knowledge on a feature *X_j_*, quantified by the change in entropy:

(7) $$\text{Gain}\left(Y,X_{j}\right)=H\left(Y\right)-H(Y|X_{j})$$

One drawback of information gain is its bias towards attributes with a large number of possible values [56]. The information gain ratio is the default splitting criterion of C4.5 and C5.0 decision trees, which attempts to correct this bias by scaling information gain with the entropy of *X_j_*:

(8) $$\text{GainRatio}\left(Y,X_{j}\right)=\frac{\text{Gain}\left(Y,X_{j}\right)}{H(X_{j})}$$

C4.5 decision tree starts modeling with the whole training set *D*, and each time chooses the attribute with maximum information gain ration to split the data set. It produces a branch/node for each value of a discrete attribute, or form a binary split for a continuous attribute. It does not stop branching until it correctly classifies all instances, or each leaf node contains a minimum number of records. Note that this split is a greedy choice and the effect of future decisions is not taken into account.

C4.5 decision tree allows the use of fields with missing values and employs post-pruning to avoid overfitting. It is capable of handling different measurement scales as well as both continuous and categorical variables. Advantages of C4.5 include easy to implement, no underlying assumptions and ability to generate non-parametric and non-linear models, and ability to reduce the dimensionality of input feature space in a way interpretable to the analysts. On the other hand, the trees produced by C4.5 are very large and complex. C5.0 is another enhancement of C4.5 with additional effectiveness, efficiencies and supports boosting. However, the C4.5 and C5.0 decision tree algorithms produce models with similar predictive accuracies [57].

While C4.5 decision trees naturally handle multiple classes, they can only examine a single feature at a time and do not perform well when the features are highly interdependent. However, they generate models that resemble the human decision-making process and become popular due to their interpretable results [58]. Examples of research papers applying C4.5 decision trees to bearing fault diagnostics include [30,48,59,60]. They have also been applied in other machinery fault diagnostics problems such as motor [61], pump [62] and shaft rotor [63].

### 2.3. Class Binarization

Real-world problems often involve multiple classes, but some classification algorithms, such as SVM, are inherently binary. Class binarization strategies reduce a k-class problem into a series of binary problems for classification. Two most common strategies in the literature are one-*versus*-one (OVO) and one-*versus*-all (OVA) [64], also named one-against-one (OAO) and one-against-all (OAA) [65]. More discussions on using binary classifiers in multi-class problems can be found in [66].

In the OVO or OAO strategy, classifiers are trained to discriminate between two of the k possible classes. One classifier is trained with a data subset of each possible pair of classes, then the output of the ( $\begin{array}{c} \text{k}\\ 2 \end{array}$) classifiers are combined for prediction. More classifiers are needed in this strategy, but the training set for each classifier is smaller and the number of instances for each class is more balanced.

In the OVA or OAA strategy, classifiers are trained to determine whether an instance belongs to one of the k classes or not. One classifier is trained with the whole training set, where each time *k* − 1 classes are re-labeled as the negative class (−1) and the unaltered class treated as the positive class. Only k classifiers are needed in this strategy, but the training set for each classifier is much larger and the number of instances for the negative class can be much larger than the positive class.

**Figure 1.**
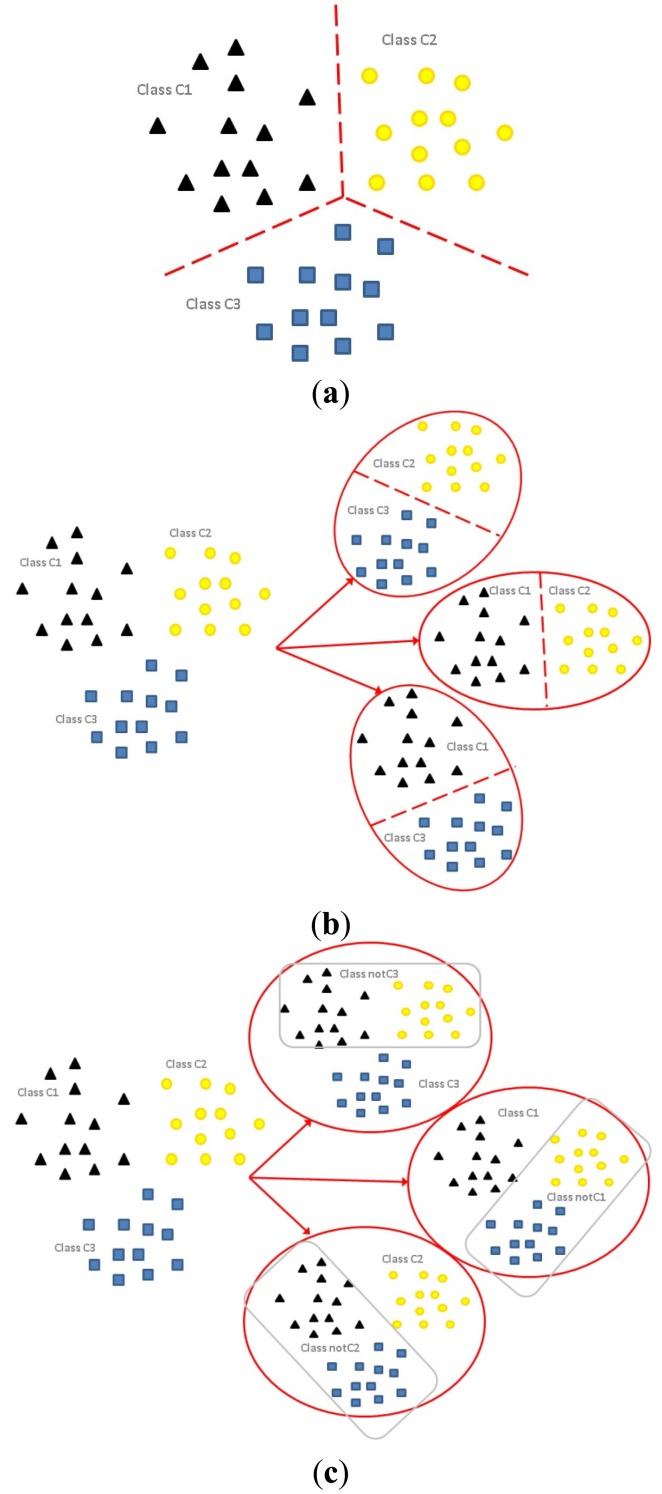
Three different classification strategies illustrated with a 3-class problem (**a**) Simple multi-class classification; (**b**) One-*versus*-one (OVO) class binarization; (**c**) One-*versus*-all (OVA) class binarization.

The differences among the formulations of multi-class classification (Figure 1a); OVO binarization (Figure 1b); and OVA binarization (Figure 1c) are illustrated graphically. For a three-class problem (*k* = 3), denote the three classes of the original problem as C1, C2 and C3. The application of the OVO binarization strategy splits the original problem into $(\begin{array}{c} 3\\ 2 \end{array})=3$ classifiers each with two classes, *i.e.*, C1 *versus* C2; C2 *versus* C3; C3 *versus* C4. On the other hand, the application of the OVA strategy (adopted in out proposed method) splits the original problem into *k* = 3 classifiers, each contains all samples with samples of *k* − 1 classes relabeled, *i.e.*, C1 *versus* notC1 (C2, C3); C2 *versus* notC2 (C1, C3); C3 *versus* notC3 (C1, C2).

These class binarization strategies can also be useful in other situations. For example, OVA class binarization strategy has been adopted in a general framework for class-specific feature selection using any feature selectors in [67]. An extensive study in [68] has shown that the two binarization strategies with an appropriate combination strategy are simple and useful ensemble methods to improve classifier performances, even when the classification algorithm itself can handle multiple classes. C4.5 decision tree used in this paper is an example of classification algorithms that can handle multiple classes.

Most of the work in binarization strategies apply multiple online empirical data sets to evaluate the classification performances in general. The authors are not aware of any work in the literature on the application of binarization strategies to the specific context of machinery fault diagnostics for our purpose. In this paper, the OVA class binarization strategy is adopted on all single-defect classes and samples of the healthy class are added to the relabeled class, to predict concurrent defects from normal and single-defect training data. This can help reduce the types of training data needed for bearing fault diagnosis of combined defects. More details of how this procedure is applied are discussed in the next section.

## 3. The Proposed Methodology

Common types of bearing defects with characteristics frequencies detectable from vibrations are: (1) fundamental train (cage) frequency (FTF); (2) ball pass frequency, outer race (BPFO); (3) ball pass frequency, inner race (BPFI); and (4) ball spin frequency (BSF). In practice, incipient faults first detected from vibration signals are usually BPFO; BPFI; or BSF.

Multiple types of defects can develop on the same bearing concurrently *i.e.*, the defect types are non-exclusive classes. From domain expert knowledge, the vibration fault signatures of multiple defects are different from that of a summation of the corresponding single defects. In other words, there are interactions between different bearing defect types and this complicates the bearing fault diagnosis process. However, in terms of problem formulation, if the interaction between defect types is small enough, combined defects can be identified from single defect classifiers, with an OVA class binarization strategy.

**Figure 2.**
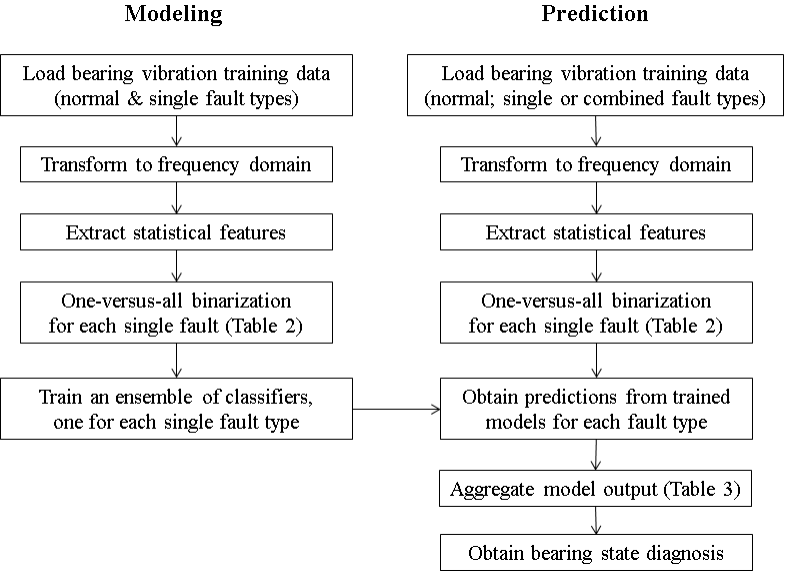
The proposed bearing fault diagnosis method with one-*versus*-all binarization.

The proposed method is summarized as a flowchart in Figure 2. Assume that the interactions between different defect types are small, the defect types can be formulated as independent non-exclusive classes. As mentioned, the OVA class binarization strategy is adopted on all single-defect classes and samples of the healthy class are added to the relabeled class. With the proposed OVA approach, a classifier can be trained to determine whether a bearing is suffering from each defect type.

As an example, consider the multi-class bearing fault diagnosis of normal and three fault types (BPFI, BPFO, BSF). Applying the proposed OVA diagnostic approach to the three fault types, the 4-class classification problem among Normal, BPFI, BPFO, BSF is transformed into three 2-class classification sub-problems. They can be denoted by classifier isBPFO that handles classes BPFO *versus* notBPFO (contains cases of types normal; BPFI, BSF); classifier isBPFI that handles classes BPFI *versus* notBPFI (normal; BPFO; BSF); and classifier isBSF that handles classes BSF *versus* notBSF (normal; BPFO; BPFI). In other words, three classifiers are trained for the three defect types BPFI; BPFO; and BSF, each using all training data of states Normal; BPFI; BPFO; and BSF.

Table 2 summarizes how the data are used in training each of the OVA binarized classifier. Note that applying the same strategy to normal cases simply generates the fault detection classifier that handles Normal *versus* notNormal.

Table 3 illustrates how outputs of three classifiers, namely isBPFO; isBPFI; isBSF, can be interpreted together to determine the state-of-health of a bearing with all possible single defects and multiple defects.

In this formulation, a normal bearing is expected to return the negative class (−1 in Section 2 or N in Table 3) for all three classifiers. Similarly, a bearing with BPFO defect is expected to generate output of the positive class from isBPFO and the negative class from isBPFI and isBSF.

If the interactions between different defect types are small enough to be ignored in modeling, a combined defect of BPFO and BPFI shall give positive for classifiers isBPFO and isBPFI, and negative for isBSF. In this case, classifiers for combined defects will not be needed and hence training data of combined defects need not be collected. On the other hand, if a combined defect cannot be correctly classified with the single-defect OVA classifiers, the OVA class binarization strategy cannot help simplify the fault diagnosis process. In this case, the interactions between different defect types have to be modeled and vibration data of different combinations of defects need to be collected, if fault diagnostics of multiple defects (which occur at a late stage of bearing degradation) is desired.

From domain expert knowledge, there is a known fixed relationship between the characteristic defect frequencies BPFO and BPFI for bearings due to geometry, *i.e.*, BPFO + BPFI (in multiple of shaft speed *S*) = number of rolling elements. It is reasonable to assume that the most interactions may be generated by vibrations due to the BPFO defect and the BPFI defect. The BPFO and BPFI combined defect is used in the subsequent empirical analyses in this paper.

## 4. Performance Evaluation—Bearing Fault Motor

In this section, the proposed OVA strategy is evaluated with experimental data from a bearing motor test bed. The SVM classifier is used as an example of statistical and data mining methods that commonly used with OVA class binarization for other purposes.

### 4.1. The Data Set

**Figure 3.**
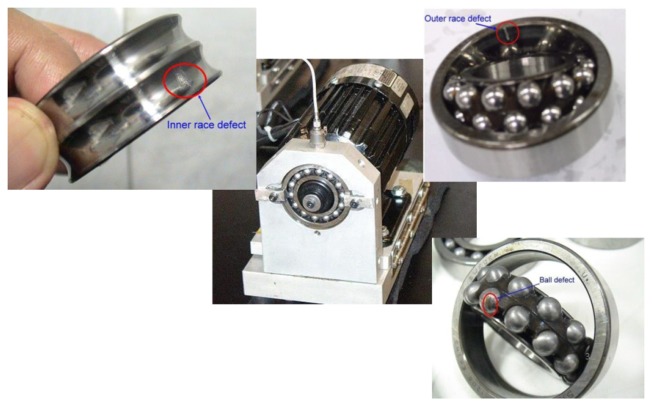
The bearing fault motor.

The data set used in this empirical analysis was collected from a bearing fault motor as shown in Figure 3. Deep groove ball bearings of model SKF 1206 were run at rotation speed of 1,400 RPM (*S* = 23.3 Hz) with no external loading. The bearing physical parameters and the bearing characteristic defect frequencies calculation are shown in Table 4.

**Figure 4.**
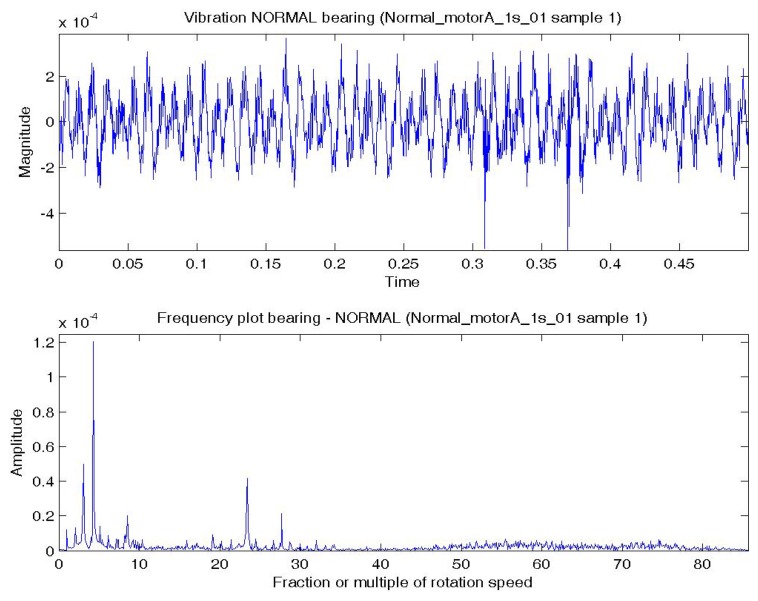
Plots of a normal bearing data sample: (**a**) Temporal signal over 0.5 s; (**b**) Spectrum—low frequency range.

**Figure 5.**
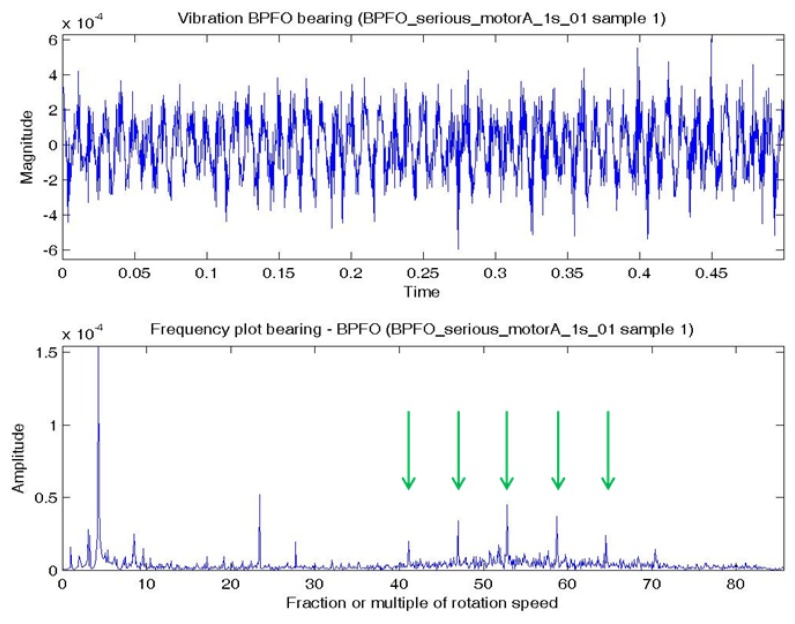
Plots of a BPFO bearing data sample: (**a**) Temporal signal over 0.5 s; (**b**) Spectrum—low frequency range.

In this experiment, the bearing could be in normal; BPFO; BPFI; BSF; or BPFO and BPFI combined state (COMB). An accelerometer was connected at vertical direction of the bearing. Vibration data were collected at a sampling rate of 4,000 Hz. Two samples were extracted for each experimental condition. Each sample consists of one temporal signal of 0.5 s length. By the Nyquist sampling theorem, the highest frequency that can be shown in a spectrum is 4,000/2 = 2,000 Hz (or 85.8 × *S*). The impulses excited by the bearing fault are most obvious by comparing a BPFO vibration data sample and normal bearing vibration data sample. Figure 4a shows a temporal signal of a normal bearing data sample and Figure 5a shows the plot of a BPFO bearing data sample. For each sample in this data set, the vibration signal is transformed by FFT to the frequency spectrum. Figures 4b and 5b show the corresponding frequency spectra of the two samples. The time between impulses in the vibration plot is the period of the defect which is the reciprocal of the characteristic defect frequency. The same information is shown as peaks at the characteristic defect frequency and its harmonics (marked with green arrows in Figure 5b. Examples that use defect characteristic frequencies and their harmonics in the data-driven modeling of bearings includes [69,70].

**Figure 6.**
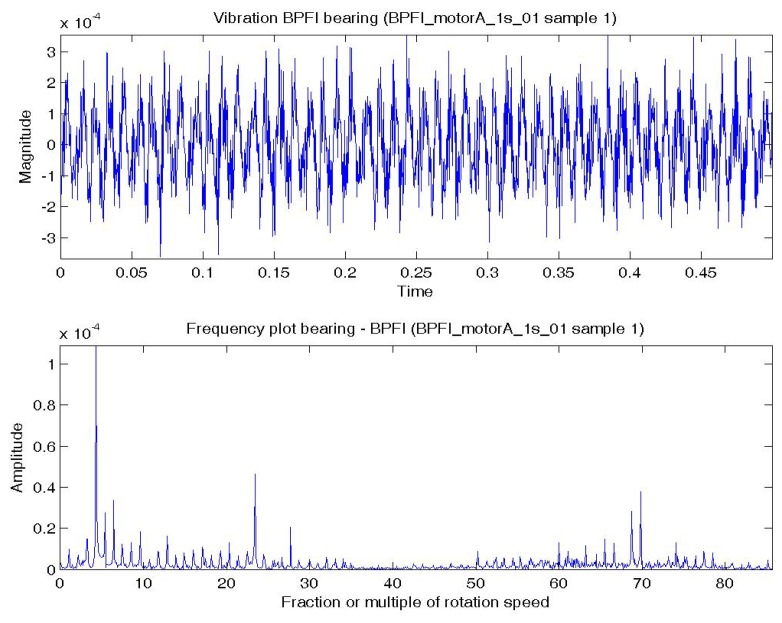
Plots of a BPFI bearing data sample: (**a**) Temporal signal over 0.5 s; (**b**) Spectrum—low frequency range.

Figure 6a shows a temporal signal of a BPFI bearing data sample and Figure 6b shows the corresponding frequency spectra. The BPFI characteristic defect frequency and harmonics are not as obvious as the BPFO, but there are numerous sidebands equal to the shaft rotation frequency (1×) in the spectrum, which are caused by modulation. The plots of BSF are less obvious to visual inspection and are omitted.

Table 5 shows the list of samples in this data set. The first eight samples were used in training and the last eight samples were used in testing. In other words, part of the bearing data collected at normal state and each of the single defect states are selected randomly for modeling training. The rest of the normal and single defect data, together with the COMB data, were used in performance evaluation of the proposed strategy.

Six summary statistics commonly used in the literature, namely median, 75-th percentile, maximum, root-mean-square (RMS), skewness and kurtosis, are extracted from the time waveform signals, the time series distributions and the frequency range between the 4th to 10th harmonics of the bearing characteristics frequencies [71]. In other words, a total of 18 features from the vibration data are used. Three classification algorithms, namely SVM, C4.5 and naive Bayes are applied with the proposed procedure and the results of the best performing method is reported below. With the use of this bearing fault motor data set, the proposed strategy is evaluated by comparing the bearing diagnostic performance with a simple multi-class formulation, as well as the random guess accuracy and the worst-case classification (classifying all cases to the majority class) accuracy.

### 4.2. Results and Discussion

The testing performance in terms of prediction accuracy of the proposed method is compared with that of a simple multi-class classification using the same data split. The confusion matrices are shown in the corresponding subsections, followed by discussions of the results. The single-defect classification performance is first compared to evaluate the improvement in diagnostic accuracy for trained (single defect) classes. The concurrent-defect classification performance is then evaluated on the potential to enable multiple-defect bearing diagnostics from single-defect training data. The diagnosis accuracy of random guess classification and worst-case classification are also considered.

#### 4.2.1. Baseline: Multi-Class Classification

In the baseline method, multiple classes are handled by pairwise classification as in typical SVM. The data are neither normalized nor standardized. Table 6 shows the prediction results of the multi-class classification with SVM. The correct fault diagnoses are highlighted in bold.

#### 4.2.2. Multiple Defects: One-*Versus*-All (OVA) Class Binarization Performance

The predictors used in the OVA formulation are the same as that in the multi-class classification. The 18 features are fed into three SVMs that model binary decisions of whether each of them belongs to each single-defect, as in Table 2. The outputs of the three SVMs are aggregated as in Table 3. Table 7 shows the results of the OVA formulation and the correct fault diagnoses are highlighted in bold.

#### 4.2.3. Performance Evaluation

The prediction performance of normal and single-defect bearings is first compared. When one-*versus*-all (OVA) formulation is used, the number of correct classification is 66.7%. This diagnosis accuracy is much higher than the 33.3% of the baseline multi-class classification, though triple computation effort is required for training. The classification accuracies of both methods are higher than the accuracy of random guess among four single-defect classes (25%).

Next we compare the performance for concurrent defects, using the COMB data. When a multi-class classification is used, there is no way for the classifier to give the correct classification because this class does not exist in the training data and hence the classifier model. When the OVA formulation is used, all of the COMB test samples (two out of two) are classified as the correct combined defect BPFI + BPFO.

The use of OVA class binarization in our proposed way increases the overall bearing fault diagnostic accuracy, from 33.3% to 66.7% for normal and single defect types, and from 0% to 100% for COMB. The overall test accuracy of 75% of the proposed method is also much better than the random guess among eight classes (12.5%) and the worst-case classification of classifying all cases to the majority class, *i.e.*, Normal (37.5%). These agree with previous studies in the literature that the use of class binarization with an appropriate aggregation strategy can help improve classification performance even when the classifier itself is capable of handling multiple classes.

For this data set, the next best performing classifier is C4.5 decision tree, which produces a classification accuracy of four out of six (66.7%) for normal and single-defect test cases in the multi-class formulation and the slightly lower three out of six (50%) in the OVA approach. However, an examination of the generated model shows that only two of the four types are modeled in the decision tree, probably because the training size is too small.

## 5. Performance Evaluation—Machine Fault Simulator (MFS)

In this section, the proposed OVA strategy is evaluated with experimental data from a multiple bearing mechanical system, which includes a shaft suspended by two bearings. The popular C4.5 decision tree is an example of statistical and data mining methods that do not normally use with binarization, yet produces high diagnostics accuracies with our proposed OVA diagnostic approach.

### 5.1. The Data Set

**Figure 7.**
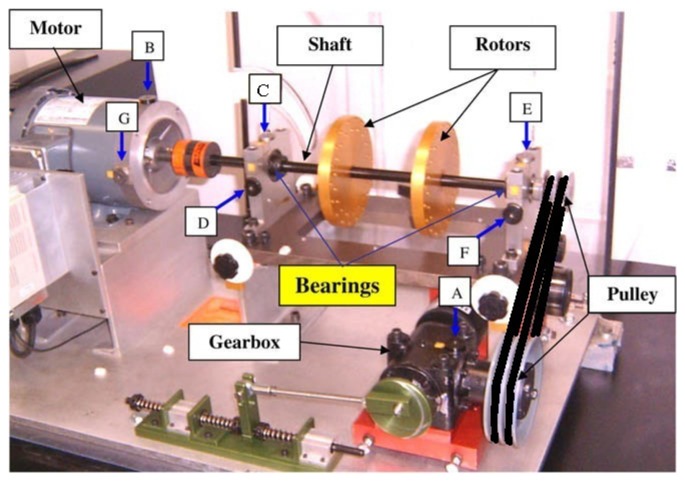
The machine fault simulator (MFS).

The data set used in this empirical analysis was collected from a machine fault simulator (MFS) as shown in Figure 7.

In this experiment, the bearing again could be in normal; BPFO; BPFI; BSF; or BPFO and BPFI combined state (COMB). Either of the bearings on the left and right of the shaft can be at fault and we assume that the faulty bearing has been located in the isolation stage. Vibration data were collected from the accelerometers at vertical and horizontal direction of the bearing either on the left or the right of the rotors, *i.e.*, either positions C and D, or E and F in Figure 7. The experiment was run at 30 Hz (1,800 RPM), with load attachment (torque 10 in-lbs or 1.13 Nm). The vibration data were collected at a sampling rate of 32,768 Hz. Ten samples were extracted for each combination of experimental conditions. Each sample consists of two temporal signals of one second length, collected from the sensors along the vertical and horizontal directions.

The total number of samples used in this analysis is: 5 (health states) × 2 (bearing locations) × 10 (replicates) = 100 observations. A 80-20 split is applied on normal and single defect data for training and testing to evaluate the prediction accuracies, *i.e.*, 80% or 64 observations of Normal; BPFI; BPFO; and BSF are used for training and 20% or 16 observations are used for testing. As in the previous experiment, all COMB data are used for testing to evaluate the performance of the trained classifier on bearing diagnostics with concurrent defects.

**Figure 8.**
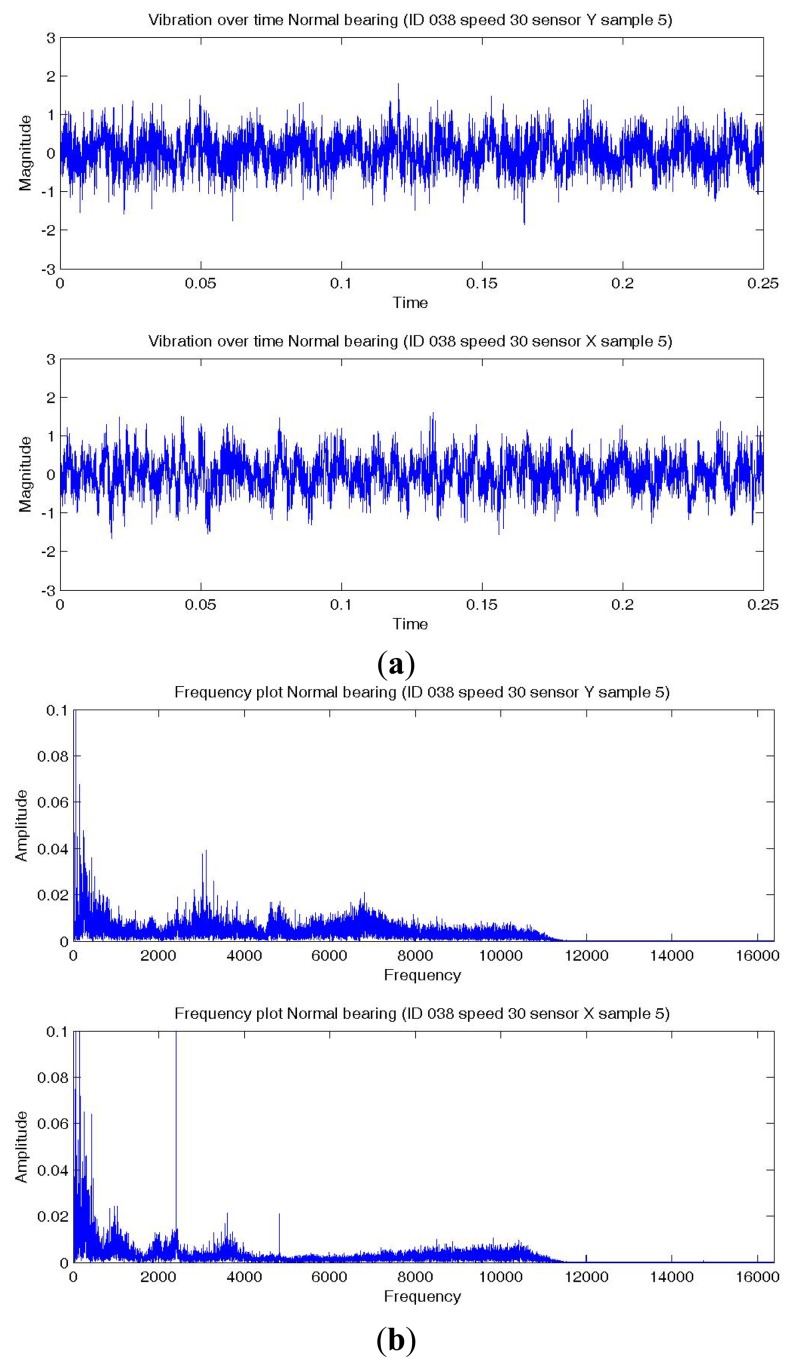
Plots of a normal bearing data sample—vertical (top) and horizontal (bottom) sensor (**a**) Temporal signal over 0.25 s; (**b**) Spectra—low and high frequency range.

**Figure 9.**
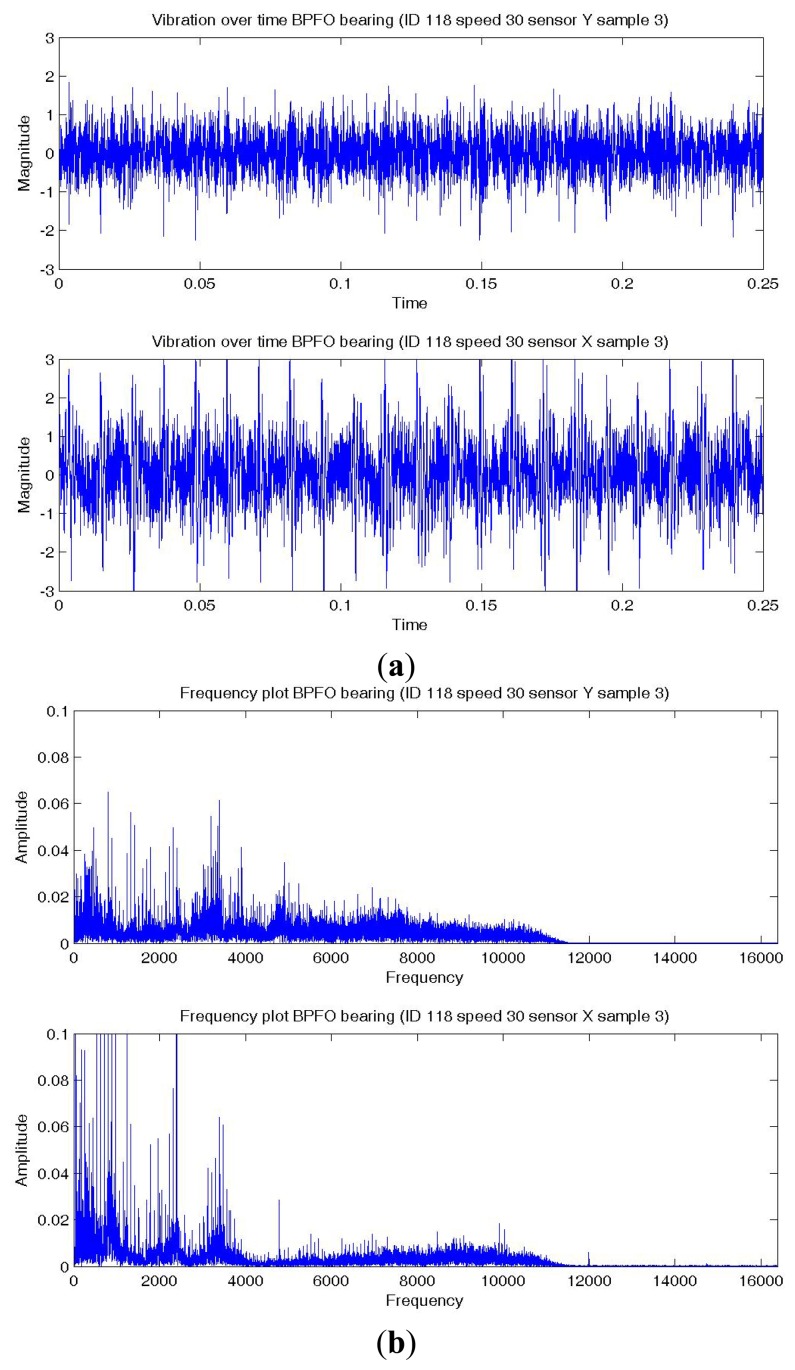
Plots of a BPFO bearing data sample—vertical (top) and horizontal (bottom) sensor (**a**) Temporal signal over 0.25 s; (**b**) Spectra—low and high frequency range.

**Figure 10.**
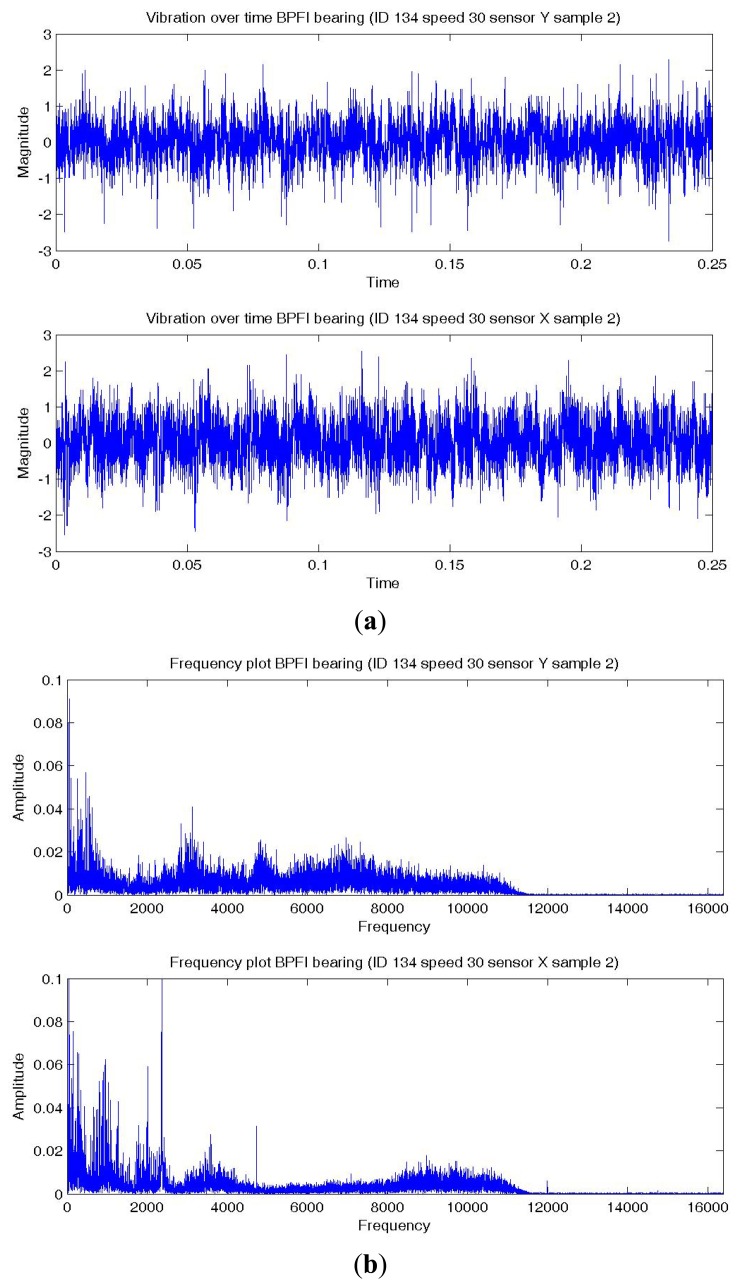
Plots of a BPFI bearing data sample—vertical (top) and horizontal (bottom) sensor (**a**) Temporal signal over 0.25 s; (**b**) Spectra—low and high frequency range.

Figures 8a,9a,10a show a temporal signal of a Normal; BPFO; and BPFI bearing data sample respectively. The two vibration signals from vertical and horizontal plane are transformed by FFT to the frequency spectra. Figures 8b,9b,10b show the corresponding frequency spectra of the three samples. Due to the much higher sampling frequency, the high frequency range (up to 16 kHz) is also captured in this data set. As shown in Figures 8b,9b,10b, the frequency range consists of a number of different distributions. Consequently, features are extracted from two frequency ranges, namely low frequency between the 4th to 10th harmonics of the bearing characteristics frequencies (Table 8), and high frequency between 7–10 kHz in the resonant frequency bands where sidebands may occur.

The same six summary statistics in the previous analysis, *i.e.*, median, 75-th percentile, maximum, root-mean-square (RMS), skewness and kurtosis, are again extracted from the time waveform signals, the time series distributions and the two frequency ranges. A total of 72 features of the vibration data from the two (horizontal and vertical) sensors are fed into C4.5 decision tree together for training and testing. Three classification algorithms, namely SVM, C4.5 and naive Bayes are applied with the proposed procedure and the results of the best performing method is reported below. With the use of this machine fault simulator data set, the proposed formulations are evaluated by comparing their bearing diagnostic performance with a simple multi-class formulation.

### 5.2. Results and Discussion

The structure of this subsection is the same as Section 4.2 for the other data set, except that the worst-case classification accuracy for this data set is the same as the random guess classification accuracy. This is because equal number of training samples is used for each bearing defect type in this empirical analysis.

#### 5.2.1. Baseline: Multi-Class Classification

Table 9 shows the prediction results of the multi-class classification with C4.5. The correct fault diagnoses are highlighted in bold.

#### 5.2.2. Multiple Defects: One-*Versus*-All (OVA) Class Binarization Performance

Table 10 shows the results of the OVA formulation and the correct fault diagnoses are highlighted in bold.

#### 5.2.3. Performance Evaluation

The prediction performance of normal and single-defect bearings is first compared. When one-*versus*-all (OVA) formulation is used, the diagnosis accuracy increases from 93.8% to 100%. The classification accuracies of both methods are much higher than the accuracy of random guess among four single-defect classes (25%).

Next we consider the performance for concurrent defects, using the COMB data. When the OVA formulation is used, eight of the 20 COMB test samples are correctly classified as BPFO + BPFI. The diagnosis accuracy increases from 0% for the baseline to 40%, also much higher than the random guess among eight classes (12.5%).

Overall, the C4.5 decision tree produces very satisfactory performances with the proposed OVA diagnostic approach. In particular, the diagnosis accuracy of combined fault samples using C4.5 with OVA is much better than that using SVM with on the same data set (which performed worse than the worst case classification accuracy on the COMB samples). This further suggests that the combined fault classification accuracy is not solely contributed by the use of polynomial kernel in the SVM.

## 6. Conclusions

In real-world applications of bearing diagnostics, multiple defect types may occur at the same time. While many data-driven modeling methods naturally handle mutually exclusive groups of data, no discussions on the handling of non-exclusive concurrent faults in bearing diagnostics are found in the literature. Moreover, the need for data collection from different operating scenarios such as permutations and combinations of fault type, location, size, machine load and speed is a concern in using data-driven methods for bearing diagnosis. In this paper, we have proposed a formulation strategy to improve diagnostics performance and reduce the number of scenarios needed in the training data, with focus on multiple defects that may occur concurrently. Using two sets of test bed data collected from a bearing motor and a multiple component mechanical system, we have shown the potential of a one-*versus*-all (OVA) approach to improve bearing diagnosis performance at the same time enable concurrent-defect diagnostics of BPFO and BPFI combined fault from normal and single-defect training data. Better formulation in bearing diagnostics helps build more practical classifiers with less data, for improved performance at more operating conditions. These help provide advanced failure warning and reduce unexpected failures in real applications. Future work includes data collection and further analysis of the proposed approach with other combined defects such as BPFI BSF combined; BPFO BSF combined; and all three types combined.

## Figures and Tables

**Table 1.** The top 10 data mining algorithms [39] and their categorization.

**DM Algorithm**	**Type**	**Formulation**
C4.5	decision tree	Classification
k-means	distance-based	Clustering
SVM (Support Vector Machine)	geometric	Classification
Apriori	rule-based	Association rules
EM (Expectation Maximation)	statistical	Clustering
PageRank	network graph	Ranking
AdaBoost	boosting	Ensemble
kNN (k Nearest Neighbor)	distance-based	Classification
Naïve Bayes	statistical	Classification
CART (Classification and Regression Tree)	decision tree	Classification; Regression

**Table 2.** One-*versus*-all binarization for three bearing defect types.

**Classifier**	**Training Data**	

	**Positive Class**	**Negative Class**
isBPFI	BPFI	notBPFI (Normal; BPFO; BSF)
isBPFO	BPFO	notBPFO (Normal; BPFI; BSF)
isBSF	BSF	notBSF (Normal; BPFI; BPFO)

**Table 3.** Fault diagnostics with a one-*versus*-all class binarization strategy.

**Classifier Output**			**Bearing State Diagnosis**

**isBPFI**	**isBPFO**	**isBSF**	
N	N	N	Normal
Y	N	N	BPFI
N	Y	N	BPFO
N	N	Y	BSF
Y	Y	N	BPFI + BPFO
Y	N	Y	BPFI + BSF
N	Y	Y	BPFO + BSF
Y	Y	Y	BPFI + BPFO + BSF

**Table 4.** Bearing parameters and characteristic frequencies (BCFs) of each defect.

**Parameter/BCF**	**Value**
No. of balls N	14
Ball diameter B	8
Pitch diameter P	46.55
Contact angle *ψ*	0
BPFI	8.203 × *S* or 115.45 Hz
BPFO	5.797 × *S* or 71.22 Hz
BSF	2.823 × *S* or 46.48 Hz

**Table 5.** Bearing fault simulation data for training and testing.

**FileID**	**Actual_Class**	**Train/Test**
BPFI_motorA_1s_01_sample_01	BPFI	Train
BPFO_motorA_1s_01_sample_01	BPFO	Train
BPFO_motorA_1s_01_sample_02	BPFO	Train
BPFO_serious_motorA_1s_01_sample_02	BPFO	Train
Normal_motorA_1s_02_sample_01	NORMAL	Train
Normal_motorA_1s_02_sample_02	NORMAL	Train
Normal_motorA_1s_03_sample_01	NORMAL	Train
ball_motorA_1s_01_sample_02	BALL	Train

BPFO_combine_BPFI_motorA_1s_01_sample_01	COMB	Test
BPFO_combine_BPFI_motorA_1s_01_sample_02	COMB	Test
BPFI_motorA_1s_01_sample_02	BPFI	Test
BPFO_serious_motorA_1s_01_sample_01	BPFO	Test
Normal_motorA_1s_01_sample_01	NORMAL	Test
Normal_motorA_1s_01_sample_02	NORMAL	Test
Normal_motorA_1s_03_sample_02	NORMAL	Test
ball_motorA_1s_01_sample_01	BALL	Test

**Table 6.** Test performance of multi-class classification of single defects.

**Actual**	**Predicted**			

	**BPFI**	**BPFO**	**BSF**	**Normal**
BPFI	**1**			
BPFO	1			
BSF	1			
Normal		2		**1**
COMB	2			

Correct				**2**

				0.333

**Table 7.** Test performance with one-*versus*-all (OVA) formulation.

**Actual**	**Overall**				

	**BPFI**	**BPFO**	**BSF**	**Normal**	**BPFO BPFI**
BPFI	**1**				
BPFO	1				
BSF				1	
Normal				**3**	
COMB					**2**

Correct				**4**	

				0.667	1.000

**Table 8.** Bearing parameters and characteristic frequencies (BCFs) of each defect.

**Parameter/BCF**	**Value**
No. of balls N	8
Ball diameter B	0.3125
Pitch diameter P	1.319
Contact angle *ψ*	0
BPFI	4.948 × *S* or 148.44 Hz
BPFO	3.052 × *S* or 91.56 Hz
BSF	1.992 × *S* or 59.76 Hz

**Table 9.** Test performance of multi-class classification of single defects.

**Actual**	**Predicted**			

	**BPFI**	**BPFO**	**BSF**	**Normal**
BPFI	**4**			
BPFO		**4**		
BSF			**4**	
Normal		1		**3**
COMB		20		

Correct				**15**

				0.938

**Table 10.** Test performance with one-*versus*-all (OVA) formulation.

**Actual**	**Overall**				

	**BPFI**	**BPFO**	**BSF**	**Normal**	**BPFO BPFI**
BPFI	**4**				
BPFO		**4**			
BSF			**4**		
Normal				**4**	
COMB	7	4		1	**8**

Correct				**16**	

				1.000	0.400
